# Root causes of first-case start time delays for elective surgical procedures: a prospective multicenter observational cohort study in Ethiopia

**DOI:** 10.1186/s13037-024-00405-z

**Published:** 2024-07-15

**Authors:** Meseret Firde, Biresaw Ayine, Getachew Mekete, Amanuel Sisay, Tikuneh Yetneberk

**Affiliations:** 1 Department of Anesthesia, Debre Tabor University, Debre Tabor, Ethiopia; 2 Department of Anesthesia, College of Medicine and Health Sciences, University of Gondar, Gondar, Ethiopia; 3 Department of Anesthesia, College of Medicine and Health Sciences, Bahir Dar University, Bahir Dar, Ethiopia

**Keywords:** Associated factors, Delay to start, Efficiency, Late start time, Operating room, Surgery, Utilization, Waste

## Abstract

**Background:**

Delays in surgery start times can lead to poor patient outcomes and considerable increases in healthcare expenditures. This is especially true in developing countries that often face systemic inefficiencies, such as a shortage of operating rooms and trained surgical personnel. With substantial effects on patient outcomes, healthcare efficiency, and resource allocation, identifying delays in first-case elective surgery is a crucial area of research.

**Methods:**

A multicenter observational study was conducted at three comprehensive and specialized hospitals in the Amhara region of Ethiopia from May 1 to October 30, 2023. The primary aim of the study was to determine the occurrence of late first-case start times, defined as a patient being in the operating room at or after the hospital’s incision time of 2:30 a.m. The secondary aim was to discover potential root causes of delayed first-case start times. All patients scheduled for elective surgery as the first case on the operating list throughout the study period were included in the study. Every emergency, day case, after-hours case, and canceled case was excluded.

**Results:**

A total of 530 surgical patients were included during the study window from May 1 to October 1, 2023. Of these, 41.5% were general surgeries, 20.4% were gynecology and obstetrics surgeries, and 13.2% were orthopedic surgery procedures. Before the procedure started, nine (1.7%) of the participants had prolonged discussion with a member of the surgical team. Patients who arrived in the operating room waiting area at or after 2:30 a.m. were 2.5 times more likely to experience a first-case start time delay than those who arrived before or at 2:00 a.m. (AOR = 2.50; 95% CI: 1.13–5.14). Furthermore, participants with abnormal investigation results were 2.4 times more likely to have a late first-case start time (AOR = 2.41; 95% CI: 1.06, 5.50). Moreover, the odds of a late first-case start time were increased by 10.53 times with the surgeon being in the operating room at or after 2:30 a.m. (AOR = 10.53; 95% CI: 5.51, 20.11).

**Conclusion:**

The research highlights a significant occurrence of delayed start times for the first elective surgical procedures. Therefore, directing attention to aspects such as ensuring patients and surgical teams arrive promptly (by or before 2:00 a.m.) and timely evaluation and communication of investigative findings before the scheduled surgery day could facilitate efforts to maximize operating room efficiency and enhance patient health outcomes.

## Background

The concept of starting the first cases scheduled for the day in each operating room (OR) on time is known as first case on-time start, and it has long been utilized as a crucial OR measure since it can forecast efficiencies within an operating day [1, 2]. It has also been shown to increase satisfaction with care, decrease unplanned cancellations, reduce workplace stress, increase the hospital’s capacity to do more elective surgeries, decrease overtime costs, increase theater productivity, and reduce patient wait times [3, 4]. All of these benefits contribute to higher-quality healthcare services and optimize the number of completed cases while ensuring patient satisfaction and maximizing utilization [5].

On the other hand, delays in the start time of the first case cause delays in subsequent cases, decreasing efficiency throughout the day [6]. The OR is a significant source of cost for hospitals because of the large volume of clinical activity, the need for numerous types of expensive equipment, and the quantity of qualified professionals needed for each case [7]. As a result, being inefficient in the OR can have serious financial consequences, as OR rooms are staffed yet underutilized [8]. In addition, during emergencies such as COVID-19 out breaks, where prompt action is essential, delays in commencing surgeries can stress already constrained resources and result in inefficiencies in patient care [9]. Moreover, delays require operating room personnel to work longer than expected, and this out-of-hours work is associated with after-hours cost increases for hospitals in the form of overtime pay [10].

Furthermore, the issue of case cancellations on the day of surgery resulting from inefficient use of theater time is widely acknowledged in hospitals [11]. A prospective descriptive study conducted at Tikur Anbessa Specialized Hospital (TASH) in Addis Ababa, Ethiopia, by Negash S et al. found that the leading cause of the cancellation rate (35.8%) was the delay in starting the first case, which led to the workday ending before scheduled cases were completed [12]. Similarly, a study from Thailand found that when the first case of the day started late, operations extended beyond the official working hours, and 10.4% of the total cancellations were last-minute actions due to insufficient time to perform the scheduled surgery [13].

Failure to begin the first procedure of the day on time frequently has a detrimental impact on the team’s overall attitude and support for efficiently completing all cases in the room [14].

Furthermore, it has been discovered that a delay in the initial case is one of the most common causes of disagreement among operating team members [15]. It has also been identified as one of the factors that lead to an unsafe working environment and increase the likelihood of staff stress and healthcare team errors [16]. Furthermore, the OR team’s anxiety and frustration with the delay may impact their subsequent performance and contribute to additional delays [17, 18].

Many surgical procedures require patients to fast in order to avoid pulmonary aspiration of stomach contents during the procedure [19]. Unfortunately, patients may find themselves waiting and fasting longer than planned on the day of the operation due to the unpredictable and inevitable delays to begin the first case on time [20]. In certain cases, failure to begin the first case on time may cause planned surgeries in the late afternoon to be canceled, requiring rescheduling and an additional round of preoperative fasting. Long-term fasting can quickly cause dehydration, which can change the way drugs work and increase the amount of fluid and blood that must be replaced during surgery and anesthesia [20, 21].

Scientific reports have also shown that patients who are waiting longer for medical care, including surgery and anesthesia, have experienced negative physiological and psychological reactions. Even waiting for health services for a few minutes can be irritating, frustrating, and a source of great uncertainty [22]. As a result, they have an increased risk of preoperative anxiety-related postoperative complications such as infection and a longer hospital stay [23, 24]. In addition, studies have found that delays in health care services are associated with low patient satisfaction [25, 26]. According to a study in Saudi Arabia, the only factor that had a significant influence on overall patient satisfaction was the delay in health service care in the hospital, with those waiting for over 30 min reporting that they were dissatisfied with the service provided [27].

Even though the start of the first case on time is considered an important factor in determining OR efficiency and quality of care, in the Ethiopian practice context, there is limited published data related to the issue, which focuses mainly on the rate and reasons for cancellation. The aim of this multicenter observational study was to investigate the prevalence of delay in starting the first case on time and assess important predicting factors. We believe that this will encourage current efforts to increase operating room efficiency, especially in terms of reducing patient waiting times for first-case start times. Furthermore, it will support upcoming research and quality-enhancement initiatives throughout Ethiopia.

## Methods

From May 1 to October 30, 2023, a prospective multicenter hospital-based observational study design was used to determine the start time of first-case elective surgeries at three public specialized and comprehensive hospitals in Amhara regional state, North West Ethiopia. The first hospital where the study was conducted was Debre Tabor Comprehensive Specialized Hospital, affiliated with Debre Tabor University and serving a population of over 2.5 million. It offers a range of surgical interventions, including general, pediatric, orthopedic, obstetrics, and gynecological surgeries, across six operating rooms. The second study setting was at Tibebe Ghion Comprehensive Specialized Hospital, located in Bahir Dar City, North West Ethiopia. Affiliated with Bahir Dar University and offers both clinical and academic services. It has over 500 beds and 11 major operating theaters for emergency and elective surgeries. The hospital provides tertiary-level surgical services across various specialties, including general surgery, neurosurgery, pediatric surgery, head and neck surgery, cardiothoracic surgery, orthopedic surgery, maxillofacial surgery, ENT (ear, nose, and throat) surgery, and obstetrics and gynecological surgery. The third study location was the University of Gondar Comprehensive Specialized Hospital in northern Ethiopia, which serves a population of more than 5 million people. Each month, the hospital performs over 520 elective surgeries, ranging from simple to complex. It has a total of 13 operating tables: 8 in the main operating room for orthopedic and various specialty surgeries, 4 for obstetrics and gynecology, and 1 for ophthalmic surgeries. These facilities were selected because they provide large clinical and academic services to a higher volume of patients, including referred cases.

The study included all elective surgical patients who were admitted to the surgical ward prior to the scheduled day of operation and scheduled as the first case on the operation list during the study period, with no restrictions on the type of surgery, anesthetic, or patient demographics. All emergency, after-hours, day cases, and canceled cases were excluded. According to the hospital’s protocol, excluding patient preparation and induction time, the incision time for the first elective case is 2:30 a.m. This start time is later compared to the typical first-case start times at hospitals worldwide. The reason for the 2:30 a.m. starting time in our study settings is the requirement for educational activities, such as morning sessions and patient rounds conducted by all surgical team departments, to be completed before the first case begins. This timing aligns with the protocol at Yale-New Haven Children’s Hospital for elective surgeries in Nigeria, where the first elective surgery begins at 7:30 a.m. on days without educational activities (Monday to Thursday) but is delayed to 8:30 a.m. on Fridays due to these activities [8].

For the purposes of this study, the late first case start time was defined as the first patient of the operating day being in the OR with fully prepared equipment, medication, and personnel at or after the incision time (2:30 a.m.). The definition is suggested after taking into account relevant studies and based on the fact that the patient must be in the operating theater with all necessary equipment, staff, and medication in order to have a skin incision on time. A semi-structured self-administered questionnaire was used to collect data related to socio-demographic characteristics (age, sex, ASA status), anesthesia characteristics (anesthesia team arrival time, if they were busy with other activities, incomplete assessment, and medications or equipment-related problems), factors relating to the hospitals’ system (a malfunctioning or lack of required materials, busy operating theaters), and other surgeon- and nurse-related factors.

Three MSc anesthetists from the study area participated in the data collection process. To ensure quality, the questionnaire was pretested on five first-case of elective surgery at Debre Tabor comprehensive specialized hospital before the actual data collection period. The collected data were imported into Epidata version 4.2 for validation purposes. The data were analyzed with SPSS version 23. Descriptive statistics were computed, and the data was analyzed using binary logistic regression. A *p*-value < 0.05 was considered statistically significant. To measure the degree of relationship between delay to start the first case and its predictors, an adjusted odds ratio with a 95% confidence interval (CI) was taken into account. Statistics such as frequencies, proportions, and summary statistics (mean, median, IQR, and standard deviation) were used to characterize the study population in relation to relevant parameters. These results were then presented in tables and graphs. Participants were informed of the study’s purpose and benefits, and they were assured that there was no risk to their safety. In addition, written consent was obtained from all participants. To preserve data confidentiality, each participant was assigned a unique code.

## Result

**Fig. 1:**
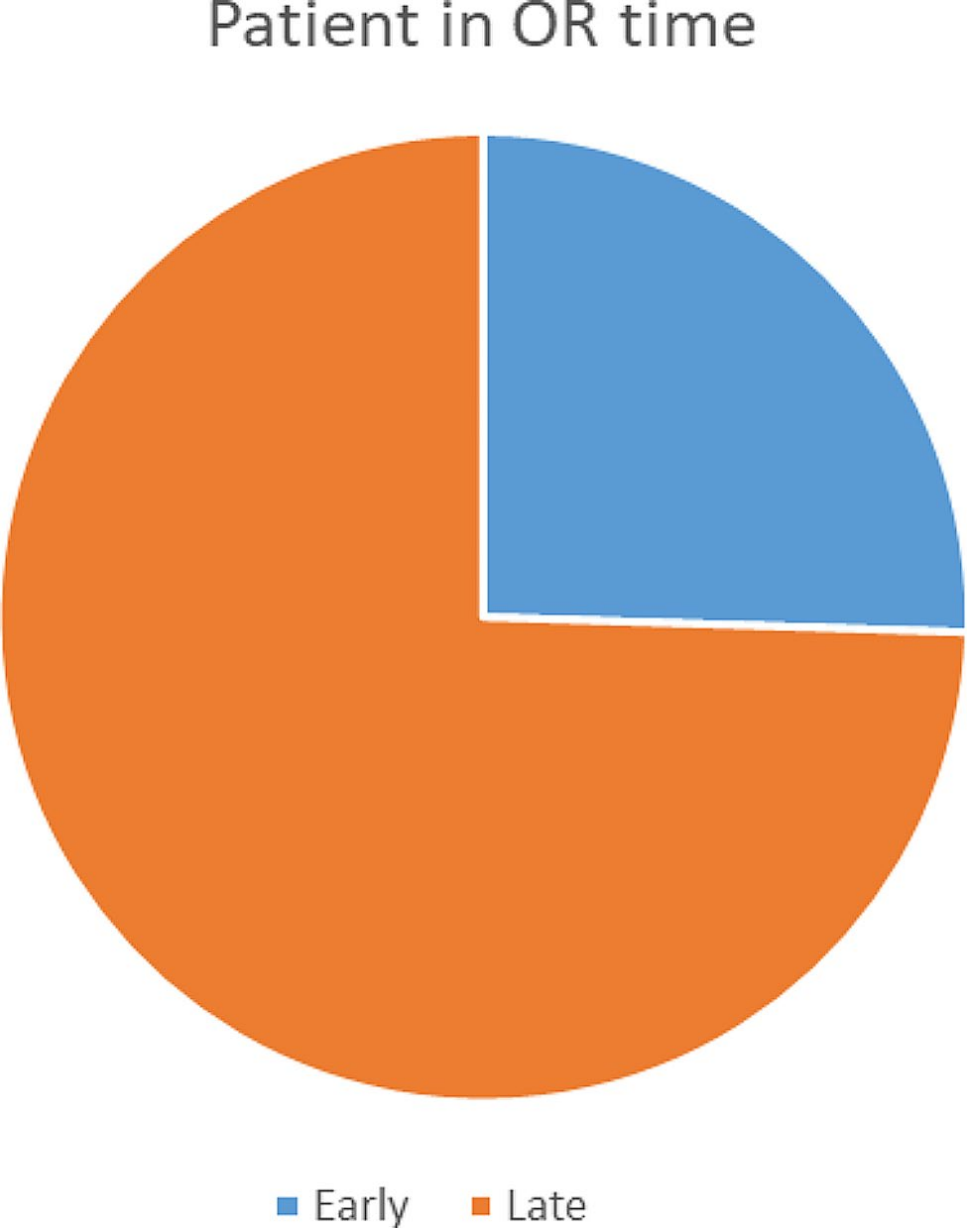
Patient being in OR time with fully prepared equipment, drug and staff

The study included a total of 530 (excluding 28 incomplete and cancelled cases) elective surgery patients scheduled as the first case of the operating day at the three hospitals where the study was carried out. The mean (standard deviation) age of participants was 34.95 (± 19.92) years. Male participants made up 325 (61.3%) of the total. The majority of study participants, 371 (70%), had American Society of Anesthesiologists status 1. All included respondents were adherent to the hospital’s null-per-oral before surgery protocol. The majority of participants (60.6%) arrived from the surgical ward to the OR waiting area at or before 2:00 a.m. (Table 1). However, only one-fourth (25.5%) of participants were physically available in OR before 2.30 am with fully prepared equipment, medication, and staff. (Fig. 1).

**Table 1:** Socio-demographic and other patient-related characteristics (*n* = 530)

Variables	Response	Frequency (*n*)	Percentage (%)
Gender	Male Female	325 205	61.5 38.7
Age	≤ 2 2–18 18–44 45–64 ≥ 65	17 85 280 94 54	3.2 16 52.8 17.7 10.2
ASA status	I II III	371 148 11	70 27.9 2.1
Waiting for family	Yes No	20 510	3.8 96.2
Patient is not adherence to preoperative fasting	Yes No	0 530	0 100
Patient is not come with ordered drugs/material	Yes No	30 500	5.7 94.3
Patient’s OR waiting area arrival time	≤ 2 2-2.3 ≥ 2.3	321 129 80	60.6 24.3 15.1

**Fig. 2:**
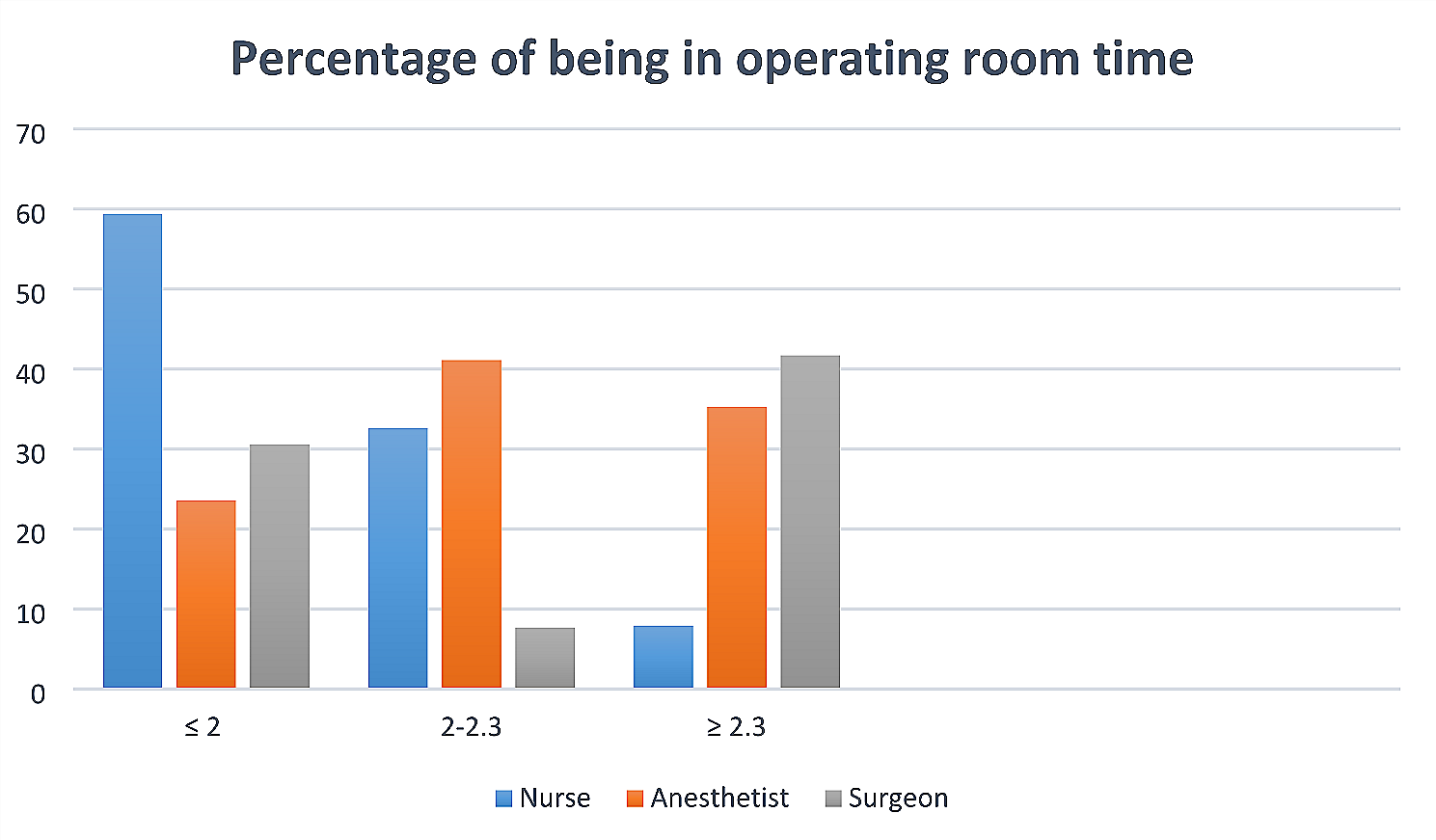
The timing of OR team being in OR

Regarding the characteristics of the anesthesia working area and anesthetists in the morning of surgery, more than half (65.5%) of the participants had general anesthesia. Incomplete evaluation by the anesthetist was revealed in 24 cases. Additionally, in 32 (6%) of cases, anesthetists missed ordering one or more orders before the day of surgery. An unavailable or malfunctioning anesthesia-related equipment problem was detected before 54 (10.2%) cases of first-case elective surgery (Table 2). Furthermore, 187 (35.28%) of anesthetists were physically present in the OR at or after 2.30 am, which is at or after the hospital’s incision time for first cases (Fig. 2).

**Table 2:** Anesthesia and anesthesia team-related characteristics (*n* = 530)

Variables	Response	Frequency (*n*)	Percentage (%)
Type of anesthesia	GA RA PNB	347 169 14	65.5 31.9 2.6
Unavailable or malfunctioning equipment	Yes No	54 476	10.2 89.8
One or more needed medications are not available	Yes No	28 502	5.28 94.72
Incomplete evaluation by anesthetist	Yes No	24 506	4.5 95.5
Missed order by anesthetist	Yes No	32 458	6.03 93.96
Anesthetists was busy with other activity	Yes No	23 507	4.3 95.7

In accordance with the findings, 220 (41.5%) of participants underwent general surgery. Additionally, 9 (1.7%) of the participants had prolonged discussions with the surgical team. Moreover, in 33 (6.2%) of cases, the surgeon was busy with other activities before the first surgery began (Table 3). In the majority of the cases, 221 (41.3%), surgeon teams were physically present in the OR at or after 2.30 am (Fig. 2).

**Table 3:** Surgery and surgeon team-related characteristics

Variables	Response	Frequency (*n*)	Percentage (%)
Type of surgery	General Urological Gyn/obs Orthopedics ENT Ophthalmology	220 67 108 70 33 32	41.5 12.6 20.4 13.2 6.2 6
Missed order by surgeon	Yes No	17 513	3.2 96.8
Incomplete evaluation	Yes No	13 507	2.45 97.55
Surgeon was busy with other activity in the morning of surgery	Yes No	33 497	6.2 93.8
Prolonged discussion with patient	Yes No	9 521	1.7 98.3

According to the findings, nearly all nursing teams were not busy with other activities before the first case of elective surgery. More than half of the responsible nurses, 315 (51%), were physically present in the OR at or before 2 a.m. on surgery day. Lack of adequate overnight preparation was detected in 33 (6.2%) of patients scheduled as first cases of elective operation days. Delayed pharmacy supply was noticed in 21 (4.1%) cases. In 26 (4.0%) of first cases, a communication gap between the surgery and anesthesia teams was recognized (Table 4).

**Table 4:** Nursing team and other hospital system-related characteristics

Variables	Response	Frequency (*n*)	Percentage (%)
Nurse team was busy with other activity	Yes No	11 519	2.1 97.9
Communication gap between the team	Yes No	26 504	4.9 95.1
Inadequate overnight preparation	Yes No	33 497	6.2 93.8
Difficulty Intravenous line in the morning of surgery	Yes No	20 510	3.8 96.2
lack or malfunctioning OR materials	Yes No	16 514	3 97
Patient given unclear instruction	Yes No	25 505	4.7 95.3
Patient with abnormal investigation	Yes No	60 470	11.3 88.7
Operating rooms are not free	Yes No	8 522	1.5 98.5
delay Pharmacy supply in the morning of surgery	Yes No	21 509	4.0 96.0

### Factors associated with late first-case start time (LFST)

Concerning factors associated with first-case delay, In the bivariate analysis, problems related to anesthesia equipment, the patient’s OR waiting area arrival time, being in OR timing of the surgeon, anesthetist, and nurse, and patient with abnormal investigation results were significantly associated with late first-case start time. However, the multivariable logistic regression model found a significant relationship between the outcome variable and patient’s arrival time, timing of surgeons to physically present in OR, and abnormal investigation result (Table 5).

**Table 5:** Factors associated with late first-case start time (*n* = 530)

Variables	**Patient in OR**		**Odds ratio (95% CI)**		Multi
					variate
	Early	Late	Crude	Adjusted	***P value***
	(n = 135)	(n = 395)			
**Anesthesia related equipment problem**					
**No**					
**Yes**	126	350	1	1	
	9	45	5.0(2.44, 10.23)*	2.16(0.99, 4.75)	0.054
**Anesthetists in OR time**					
**≤ 2**					
**2-2.3**	44	81	1	1	
**≥ 2.3**	52	166	3.19(2.34, 4.36)*	1.45(0.96, 2.20)	0.07
	39	148	3.79(2.67, 5.40) *	1.11(0.68, 1.81)	0.67
**Surgeon in OR time**					
**≤ 2**	67	95	1	1	
**2-2.3**	54	93	1.72(1.23,2.41)*	1.11(0.70, 1.76)	0.64
**≥ 2.3**	14	207	14.78(8.61,25.4)*	10.53(5.51,20.11)*	0
**Nurse in OR time**					
**≤ 2**	87	228	1	1	
**2-2.3**	39	134	3.44(2.41,4.91)*	0.72(0.43,1.22)	0.22
**≥ 2.3**	9	33	3.67(1.75,7.66)*	1.05(0.45,2.47)	0.9
**Patient arrival time to OR waiting area**					
**≤ 2**					
**2-2.3**	96	225	1	1	1
**≥ 2.3**	28	101	3.61(2.37, 5.48)*	1.28(0.76, 2.17)	0.37
	11	69	6.27(3.32,11.85)*	2.5(1.13, 5.14)*	0.023
**Abnormal investigation**					
**No**					
**Yes**	127	343	1	1	0.037
	8	52	6.5(3.09, 13.68)*	2.41(1.06, 5.50)*	

## Discussion

Only 25.5% of participants were physically present in the OR before the hospital’s incision time of 2.30 a.m., with staff, equipment, and medication ready, despite the majority of participants (60.6%) arriving at the OR waiting area at or before 2.00 a.m. This implies that even with prompt arrivals from the surgical ward, there appears to be a delay in the operating room’s preparation. Furthermore, this signifies that there may be room for improvement in terms of streamlining procedures and making sure that the required personnel levels and resources are ready and available for incoming patients.

The study also revealed that 74.5% of first-case patients entered the operating theater at or after the incision time, indicating a delayed first-case start time. This finding is comparable to a nine-month retrospective analysis of a related study from 22 German hospitals, where 70% of first-case start times were seen to be delayed [2]. In contrast, our findings indicated a higher frequency of late first-case start times when compared to related studies by Mathews et al. and Kayla B. Hicks et al. where the incidence of first-case delay was reported to be 67% and 55%, respectively [28, 29]. Our findings also revealed a lower incidence of first-case delays compared to a study from Addis Ababa, Ethiopia, where the start time was delayed in 93.4% of cases [12]. Similarly, a 15-month prospective study conducted in Nigeria reported first-case delays in almost all patients at the study center, with a rate of 99.3% [30]. Varying definitions of first case delay, study setting, study population, and causes for delay could be the cause of this large variation in prevalence. There are consequences for OR efficiency, patient care, and expenditure of resources from this occurrence of late first case start times, which represents a serious challenge to perioperative management.

Additionally, incomplete preoperative anesthetic evaluation was observed in 24 (4.5%) of the cases. In order to identify any patient-related problems or concerns that might affect the surgical procedure’s schedule or operation, the anesthetist must perform a thorough review. If these concerns are not addressed during the preoperative evaluation, there may be last-minute modifications or interventions, which could cause the first case to start later than expected [31]. Additionally, insufficient assessment may result in the need for extra time to retrieve or prepare patient-specific materials or drugs, which would further delay the start of the surgical procedure [32].

Coordinating and communicating amongst members of the surgical team can also be hindered by the anesthetist’s missing orders. In 32 cases (6%), anesthetists forgot to make a preoperative order for one or more pieces of equipment or drugs. Partially prepared preoperative care might occur when an anesthetist neglects to order supplies or drugs required before the day of surgery for a surgical procedure. Thus, the beginning of the first case may be delayed since the surgical team has to spend more time obtaining or getting ready for these supplies [33].

Furthermore, a problem with anesthesia-related equipment was discovered prior to 54 (10.2%) of first-case elective surgeries. It is commonly understood that anesthesia professionals rely on a variety of equipment, including anesthetic machines, monitors, and airway management devices, to ensure patient safety and optimal anesthesia delivery. When anesthesia-related equipment is unavailable or malfunctioning, this may require efforts to repair or replace equipment, obtaining alternate equipment from other departments or facilities, or adjusting the surgery schedule to accommodate the delay. These resource-intensive steps can waste significant time and manpower, compounding delays in initiating the initial case on time [34].

Patients scheduled for surgery often have overnight pre-operative preparations that must be completed before the procedure. A lack of proper overnight preparation was discovered in 33 (6.2%) of patients scheduled for the first day of elective surgery.

This finding is supported by an audit to assess different perspectives of the OR staff regarding the various causative factors of first-case operative delay in the OR, which revealed that more than 60% of respondents cited a lack of adequate preoperative patient preparation as the reason for the surgery start-time delay [1]. Inappropriate or inadequate overnight preparation has also been determined to result in delays in locating and retrieving necessary items and needed medication, further contributing to the delay in starting surgery [35].

Furthermore, before the first case of the operation day, the surgeon was preoccupied in 33 (6.2%) of the cases with other activities such as the patient consultation administrative work, and other clinical responsibilities. Efficient time management and prioritization are necessary to strike a balance between these tasks and make sure that the first case of the day gets to the operating room on time. On the other hand, if morning rounds or other activities extend beyond the first case’s scheduled start time, the surgeon’s entrance into the operating room may be delayed, which may result in later first case start times [7, 33].

First-case surgical procedures cannot start on schedule without the presence of the entire surgical team, which includes surgeons, surgical assistants, anesthesiologists, nurses, and other support staff [36]. In the majority of cases, surgical teams (surgeons or assistants) and anesthetists were physically present in the OR at or after 2.30 a.m., which is after the hospital’s first-case incision time. Anesthetists require sufficient time before surgery in order to prepare the anesthetic workstation, go over patient data, and review preoperative evaluations. If they are late, it could interfere with workflow and teamwork, delaying the initial case [7, 34, 37]. Similarly, surgeons and support staff need sufficient time to review patient charts, gather necessary equipment, and conduct final assessments before the start of surgery. Delays in these preparations due to late team arrival can prolong the overall surgical timeline and contribute to late first case start times [2, 38].

The first case start time delay was approximately 2.5 times more likely to occur for patients who enter the OR waiting area from the ward at or after 2:30 a.m. than for those who arrive before or at 2:00 a.m. An observational study of 889 cases by Chinonye et al. found that the main cause of the delay in the first case was the porters’ tardiness. A related study indicates that the primary reason for the theater’s inefficiency in starting surgery late was the patient’s delayed arrival in the operating room [39, 40]. Chekol et al. from Debre Tabor, Ethiopia, also discovered that the common cause of scheduled case cancellations was inadequate patient preparation before the morning of surgery [41]. This could be explained by arriving early allows sufficient time for preoperative preparation, including patient assessment, consent procedures, preparing the operating table and anesthesia induction. Early arrival also allows for thorough communication between the surgical team, anesthesia providers, and other healthcare professionals involved in the procedure [22, 42].

This study discovered a significant association between the first case’s delayed start time and the surgeon’s actual arrival time in the operating room. Thus, there was a 10.5-fold increase in the chance of a delayed first-case start time if the surgeon entered the operating room at or after 2:30 a.m. Comparably, a study conducted in South Africa and India demonstrated that surgeon-related factors, including late surgeon arrival time, were the first reasons for the first-case start time delay [4, 43]. Anesthetists and surgeons’ tardiness was found to be the primary cause of late surgery start times in a baseline assessment of an interventional study from Rwanda that aimed to increase the percentage of first surgeries starting on time from 3–25% [7]. This could be explained by the fact that surgeons are essential to the preoperative stages of the procedure; they organize surgical sites, update patient assessments, and collaborate with anesthesia providers, nurses, and support staff to ensure that everyone is ready for the scheduled surgery. When a surgeon is late, these crucial steps get started later, which results in later first-case start times.

Furthermore, patients who arrived with abnormal investigation results were 2.4 times more likely to have a delayed first case start time. Our result is supported by several suggestions, despite the fact that it does not align with those of other relevant studies. In order to assess the clinical relevance of abnormal laboratory data and their possible influence on the surgical process, more investigation may be necessary [44, 45]. Extra consultations or permissions from other healthcare practitioners, such as specialists or medical consultants, may also be necessary in situations where abnormal investigation results raise concerns about a patient’s health or fitness for surgery. Furthermore, considering the patient’s underlying medical issues, the surgical team may evaluate the advantages and disadvantages of moving forward with surgery in response to abnormal investigation results. The extra evaluation, conversation, and coordination of consultations can all interfere with the scheduled procedure and cause delays in the commencement of surgery [15, 45].

### Limitation

The study’s strengths include the fact that it is multicenter and prospective in nature, with the aim of identifying root causes of first-case delay by taking into account more factors, including patient characteristics such as age and comorbidities, which improves the findings’ applicability to larger populations or healthcare settings. Despite this, the study may have various limitations. First, due to the fact that patient preparation, induction, airway management, and regional or peripheral nerve blocks may take some time after the patient enters the operating room, our definition of late first-case start time may not accurately reflect the actual incidence of late first-case start time. Likewise, we also only focus on pre-operative time waste before the patient enters the OR, and we do not assess time waste in the OR before starting the incision. Second, we only examined the late start time of first-case surgery and did not include wasted time during other phases of surgery, such as turnover time. The fourth limitation is that data on first-case delays was collected only by anesthetists, which may reflect a reporting bias and alter the study’s outcome. As a result, future studies that take into consideration the limitations of this study are recommended.

## Conclusion

The research findings suggest that nearly two-thirds of first-case patients experience delays in starting their procedures, which can result in inefficient utilization of OR resources and contribute to patient anxiety and dissatisfaction with their surgical experience. Implementing measures to improve patient arrival times to the operating room waiting area by 2:00 a.m., addressing late availability of surgical teams, and ensuring timely evaluation and communication of investigative results by the relevant team before the scheduled surgery day may aid in reducing the frequency of delayed first-case start times, promoting optimal use of operating room facilities, and enhancing patient health outcomes.

## Data Availability

No datasets were generated or analysed during the current study.

## Abbreviations

OR: Operating room
